# Mucosal-Associated Invariant T Cells Display a Poor Reconstitution and Altered Phenotype after Allogeneic Hematopoietic Stem Cell Transplantation

**DOI:** 10.3389/fimmu.2017.01861

**Published:** 2017-12-21

**Authors:** Martin Solders, Tom Erkers, Laia Gorchs, Thomas Poiret, Mats Remberger, Isabelle Magalhaes, Helen Kaipe

**Affiliations:** ^1^Department of Laboratory Medicine, Karolinska Institutet, Stockholm, Sweden; ^2^Blood and Marrow Transplantation, Stanford University School of Medicine, Stanford, CA, United States; ^3^Center for Allogeneic Stem Cell Transplantation, Karolinska University Hospital, Stockholm, Sweden; ^4^Department of Oncology-Pathology, Karolinska Institutet, Stockholm, Sweden; ^5^Clinical Immunology and Transfusion Medicine, Karolinska University Hospital, Stockholm, Sweden

**Keywords:** allogeneic hematopoietic stem cell transplantation, mucosal-associated invariant T cells, immune reconstitution, graft-versus-host disease, sirolimus, PD-1, cyclosporin A

## Abstract

Mucosal-associated invariant T (MAIT) cells are innate-like T cells which are important in the defense against certain bacteria and yeast. The reconstitution of MAIT cells after allogeneic hematopoietic stem cell transplantation (HSCT) is not known. We investigated MAIT cell phenotype and function in 17 patients devoid of relapse and severe graft-versus-host disease (GvHD) in paired samples collected 1–2, 3–6, 12, and 24 months after transplantation. Data were compared to 17 healthy controls (HC), as well as 22 patients with acute GvHD grade 2–3. The frequency of MAIT cells within CD3^+^ cells was approximately 10-fold lower than in HC and did not increase over the 2 years following HSCT. MAIT cells in HSCT patients displayed an elevated expression of CD69 and intracellular granzyme B and were predominantly composed of CD4/CD8 double-negative cells. The expression of PD-1 on MAIT cells was low and did not change during the observational time, whereas the CD3^+^CD161^dim/neg^TCRVα7.2^dim/neg^ cells (non-MAIT T cells) displayed a high expression early after HSCT that decreased to normal levels at 24 months. MAIT cells collected 2–6 months post-HSCT showed an impaired IFN-γ and perforin response after bacterial stimulation, but the response was restored at 24 months. Patients with acute GvHD had similar proportions of MAIT cells as patients with grade 0–1, but consisted mainly of CD8^+^ cells. Finally, MAIT cells were more sensitive to cyclosporine A and sirolimus than non-MAIT T cells. To conclude, MAIT cell reconstitution following HSCT is deficient compared to non-MAIT T cells and GvHD grade ≥2 is not correlated with MAIT cell frequency. MAIT cell functionality was impaired early after HSCT, but restored at 24 months post-HSCT. MAIT cells have an increased sensibility to common immunosuppressive drugs, which maybe could explain their hampered reconstitution after HSCT.

## Introduction

Allogeneic hematopoietic stem cell transplantation (HSCT) is an established treatment for hematological malignancies (1, 2), immunodeficiencies, and some inborn errors of metabolism (2, 3). Using HSCT as immunotherapy against cancer, the graft-versus-leukemia (GvL) effect needs to be balanced against the risk of graft-versus-host disease (GvHD) (4–6). The immune system of the patient has to be rebuilt following HSCT. The innate immune system is reconstituted during the first months after HSCT, but it takes years to develop a competent adaptive immune system (7). During this time, the patients are at higher risk of acquiring opportunistic infections.

T cells from the transplanted graft are a vital part of the adaptive immune cell compartment of HSCT patients during the first year after transplantation, although with a reduced TCR repertoire diversity (8). Non-hematological cells produce IL-7 and IL-15 at homeostatic levels, but as cytokine consumption declines following HSCT, plasma levels rise (8). Under the influence of this cytokine excess and available antigens, T cells from the graft can expand (9) and provide a base level for the adaptive immune system until T cells are generated *de novo* (10, 11). New T-cells differentiate from the transplanted stem cells in measurable amounts after approximately 3 months following HSCT (10). The amount of naïve T cells in transplanted patients is linked to thymic function, and as thymic output decreases with age, the reconstitution of T cells in adult patients is poor compared to children (12). If the patients experience complications such as GvHD, relapse or infection by LPS-producing bacteria, or CMV, the immune reconstitution is hampered further (11, 13–15).

Mucosal-associated invariant T (MAIT) cells are a subset of innate-like T cells with a potent capacity to respond to bacterial antigens. MAIT cells are activated by vitamin B metabolites (16) presented by the non-classical MHC class I related molecule (MR1) (17). MAIT cells are characterized by the expression of the invariant TCRα chain Vα7.2-Jα33 and the C-type lectin CD161 (18). The majority of MAIT cells are CD8^+^ T cells, but can also be CD4/CD8 double negative (DN) or CD4^+^. MAIT cells responds to riboflavin metabolizing microbes, including *Escherichia coli, Mycobacteria, Lactobacillus* species, and yeast (19). They can be functionally activated in a MR1-independent manner by inflammatory cytokines (20, 21), and thus promote antiviral responses (22). MAIT cells elicit their function by secreting IFN-γ, tumor necrosis factor-α (TNF-α), and IL-17 (19, 23), and by lysing infected cells after production of cytotoxic molecules, such as granzyme B (GrzB) and perforin (24, 25). MAIT cells are dependent on a functional thymus for their development (26), and they divert from the maturation steps of CD3^+^CD161^dim/neg^TCRVα7.2^dim/neg^ cells (non-MAIT T cells) when they are still double positive for CD4 and CD8 (26, 27). MAIT cells are relatively abundant in peripheral blood, representing up to 10% of all T cells, but they have been found to be enriched in mucosal tissues and liver (23). Germ-free mice lack MAIT cells, at least locally in the lamina propria and mesenteric lymph nodes, indicating that a commensal flora is necessary for a normal development of MAIT cells (17).

Despite their importance in antibacterial defense, the reconstitution and function of MAIT cells following HSCT have not been studied previously. By analyzing paired consecutive blood samples up to 2 years after HSCT, we aimed at investigating the reconstitution of MAIT cells, as well as their functionality following HSCT. Interestingly, we found that MAIT cell reconstitution was poor compared to non-MAIT T cells, but that their functionality was gradually restored.

## Materials and Methods

### Patients

Patient characteristics are summarized in Table 1. Blood samples were collected prospectively from HSCT patients from 2010 to 2016, resulting in a total inclusion of 262 patients, who were at least two years after HSCT, with varying availability of samples. Adult patients from this cohort were selected based on a global acute GvHD grade of 0–1, no more than mild chronic GvHD, no relapse during the first 24 months, not missing more than 2 out of 6 samples, and availability of the sample at 24 months post-transplantation. Based on these criteria, 17 patients were found and included. Data on 22 patients with an overall acute GvHD grade of 2–3, all with gut involvement, was added from another cohort. The median time from HSCT to GvHD symptoms were 81 days (range 8–375), HSCT to start/increase of corticosteroids were 84 days (range 8–382), and HSCT to sample collection 100 days (range 15–406). At the time of sample collection, all patients received ≥1 mg/kg/day prednisone equivalents of corticosteroids. One of these patients developed GvHD symptoms following a retransplantation, one following donor lymphocyte infusions, and one following first a retransplantation and then donor lymphocyte infusions. The GvHD grade 0–1 group received a higher CD34 dose and had higher grades of chronic GvHD. However, the chronic GvHD developed later and was not present at the time of sample collection. Apart from this, there were no other significant differences between the groups of transplanted patients (Table 1). All patients received ciprofloxacin from day −1 and during the aplastic phase of the transplantation. In addition, trimethoprim/sulfamethoxazole was administered both during conditioning and from before discharge until approximately 6 months after transplantation. Peripheral blood mononuclear cells (PBMCs) from 17 healthy blood donors were also analyzed and referred to as healthy controls (HC). These HC were anonymous, and no characteristics were available.

**Table 1:** Patient characteristics.

	Study population	GvHD population	*p* Value
*n*	17	22	
Age	56 (33–72)	53 (10–72)	0.148
Sex (female/male)	5/12	8/14	0.740
Diagnosis			0.945
Acute leukemia	6	6	
Chronic leukemia	2	2	
Lymphoma	2	3	
MDS/MPN	6	8	
Myeloma	0	1	
Non-malignant	1	2	
Stage (early/late)	6/11	7/15	1.000
Donor (SIB/MUD/Twin/Haplo)	2/14/1/0	9/12/0/1	0.108
BM/PBSCs	1/16	3/19	0.618
CD34^+^ cell dose (×10^6^/kg)	7.7 (1.7–11.6)	6.0 (1.4–13.3)	0.049
GvHD prophylaxis: CsA + MTX/Tac + Sir/PT Cy	11/4/1	14/7/1	0.888
MAC/RIC	6/11	6/16	0.73
TBI/chemo based	3/14	7/15	0.464
ATG (yes/no)	13/4	13/9	0.318
aGvHD (0/I/II/III)	11/6/0/0	0/0/3/19	<0.0001
cGvHD (none/mild/moderate/severe)	15/2/0/0	9/9/3/1	0.024

*MDS, myelodysplastic syndrome; MPN, myeloproliferative neoplasm; SIB, sibling donor; MUD, matched unrelated donor; BM, bone marrow stem cells; PBSCs, peripheral blood stem cells; CsA + MTX, cyclosporine A + methotrexate; Tac + Sir, tacrolimus + sirolimus; PT Cy, post-transplant cyclophosphamide; MAC, myeloablative conditioning; RIC, reduced intensity conditioning; TBI, total body irradiation; ATG, anti-thymocyte globulin; GvHD, graft-versus-host disease*.

### Data Handling

**Figure 1:**
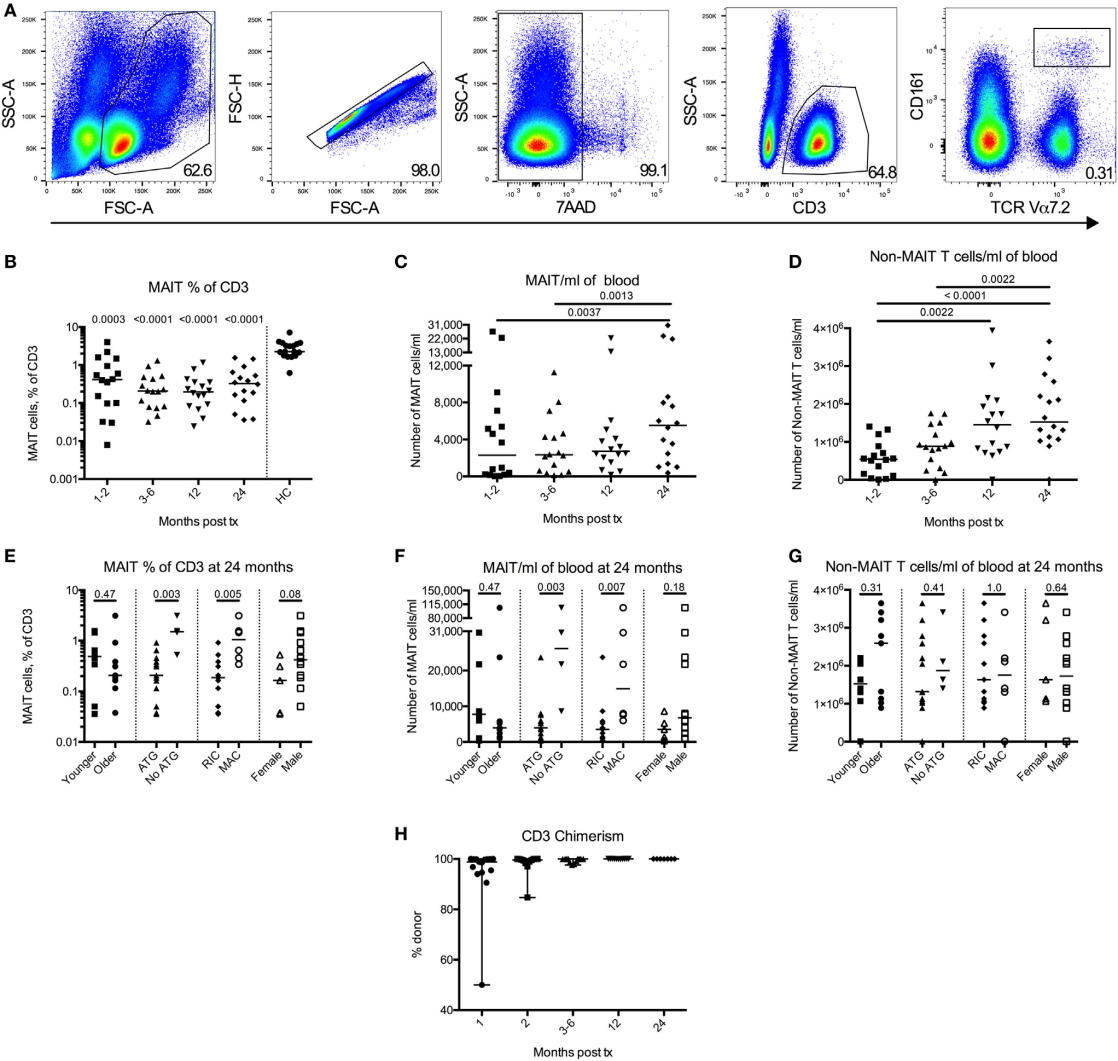
Mucosal-associated invariant T (MAIT) cells have a delayed reconstitution pattern compared to non-MAIT T cells. **(A)** Representative flow cytometry plots from one patient showing the gating strategies to identify MAIT cells. **(B)** MAIT cells expressed as percentage of CD3^+^ cells measured at four time points after hematopoietic stem cell transplantation (HSCT) (*n* = 16). Data from healthy controls (HC) are shown to the right of the dotted line (*n* = 17). Estimated number of **(C)** MAIT cells/ml of blood and **(D)** CD3^+^CD161^dim/neg^TCRVα7.2^dim/neg^ cells (non-MAIT T cells)/ml of blood measured at four time points after HSCT (*n* = 16). **(E)** MAIT cell frequency, **(F)** number of MAIT cells/ml blood, and **(G)** number of non-MAIT T cells/ml of blood at 24 months divided into and compared between groups [Younger (<56 years, *n* = 8) versus older (≥56 years, *n* = 9), anti-thymocyte globulin (ATG) (*n* = 13) versus no ATG treatment (*n* = 4), reduced intensity conditioning (*n* = 11) versus myeloablative conditioning (*n* = 6) and female (*n* = 5) versus male (*n* = 12)]. **(H)** Donor chimerism on CD3^+^ cells in peripheral blood measured at 1 (*n* = 15), 2 (*n* = 12), 3–6 (*n* = 14), 12 (*n* = 11), and 24 months (*n* = 7) after HSCT, expressed as percentage of donor DNA. Horizontal lines indicate the median value. Comparisons were made using the Friedman test followed by Dunn’s post-test between the four time points after HSCT. The Kruskal–Wallis test followed by Dunn’s post-test was used to compare patients with controls. In panel **(B)**, numbers above data points indicate *p*-values for comparisons with HC. Comparisons between different groups in **(E–G)** were made using the Mann–Whitney test.

Mucosal-associated invariant T cell reconstitution after HSCT was investigated in 17 patients at approximately 1, 2, 3, 6, 12, and 24 months post HSCT. Eight of the sample series were complete, whereas the other nine series had one or two samples missing. To perform repeated measures statistics, we consequently pooled the data, using the mean result if there were two samples available, and the data are presented as time points 1–2, 3–6, 12, and 24 months. For one of the patients, the sample at 12 months was missing, and hence the cells from this patient was not included in the reconstitution analysis, but used for other comparisons (Figures 1E–H, 3, and 4; Figures S1, S3, and S4A in Supplementary Material).

To calculate the number of CD3^+^ T cells and MAIT cells per ml of blood, data on routine clinical blood counts (neutrophil count subtracted from total leukocyte count) from the same day or as close as possible were collected from the patient charts. Based on these parameters, an estimate could be made on how many cells in the blood of the patients were represented by each cell analyzed by flow cytometry. Data were not available to make these calculations for the HC samples, since it was derived as buffycoats from the blood bank.

### Flow Cytometry

All experiments were performed using PBMCs previously isolated from blood and stored in liquid nitrogen until use. PBMCs from HC were handled the same way. The following antibodies were used: phycoerythrin (PE)-cyanine 7 (PE-Cy7), FITC or V450-conjugated anti-CD3 (UCHT1), V500-conjugated anti-CD4 (RPA-T4), Alexa Fluor 700-conjugated anti-CD8 (RPA-T8), FITC or Pe-Cy7-conjugated anti-CD69 (L78), Brilliant Violet 421-conjugated anti-CD279 (PD-1) (EH12.1), PE-Cy7-conjugated anti-IFN-γ (4S.B3), FITC-conjugated anti-granzyme B (GB11), and Alexa Fluor 647-conjugated anti-perforin (δG9; all from BD Biosciences, Franklin Lakes, NJ, USA), PE-conjugated ant-CD161 (HP-3G10), Allophycocyanine-indo tricarbocyanine (APC-Cy7)-conjugated anti-TCR Vα7.2 (3C10; both from BioLegend, San Diego, CA, USA), and APC-conjugated anti-CDw199 (CCR9) (112509; R&D Systems, Minneapolis, MN, USA). Intracellular staining was performed subsequent to extracellular staining using the BD Cytofix/Cytoperm™ kit (BD Biosciences) according to the manufacturer’s instructions. When only analyzing extracellular markers, 7AAD was used to exclude dead cells. Data were collected using a BD FACSCanto flow cytometer and analyzed with FlowJo software (Tree Star, Ashland, OR, USA). Sub-gating was only performed when the parent gate contained ≥100 events.

### Bacterial Stimulation Assays

In seven of the patients, enough cells were available to perform functional assays. At the time of sample collection, none of these patients had fever or any other sign of bacterial infections. PBMCs were incubated at a concentration of 7.5 × 10^5^ cells in 250 μl/well in RPMI medium (HyClone, GE Health Sciences, South Logan, UT, USA) supplemented with 10% FCS, 100 U/ml penicillin, and 100 µg/ml streptomycin (complete medium). The *E. coli* strain used in this study was a clinical isolate of a resident strain from fecal samples from a child (28). The bacteria were fixed in 1% paraformaldehyde and were added to the cultures at a multiplicity of infection of 40 together with 1.25 µg/ml anti-CD28mAb (CD28.2, Biolegend, San Diego, CA, USA). After 12 h of culture at 37°C and 5% CO_2_, 10 µg/ml Brefeldin-A (Sigma-Aldrich, St. Louis, MO, USA) was added, followed by an additional 4 h of culture. Supernatants were collected at the end of the culture and frozen at −80°C. Cells were then harvested, washed, and stained for flow cytometric analysis.

### ELISA

For analysis of IFN-γ and GrzB in culture supernatants, ELISA kits (Mabtech, Stockholm, Sweden) were used according to the manufacturer’s instructions.

### CFSE Proliferation Assay

To examine the anti-proliferative effect of cyclosporine A (CsA) and sirolimus (Sir) on MAIT and non-MAIT T cells T cells, PBMCs from HC were labeled with CFSE (Thermo Scientific, Waltham, MA, USA) for 15 min at 37°C in phosphate-buffered saline supplemented with 0.2% bovine serum albumin. After washing, cells were resuspended in complete medium at 2 × 10^6^ cells/ml, stimulated with the anti-CD3 (OKT-3, Biolegend) antibody (25 ng/ml) or left unstimulated. CsA (10, 100, 500, or 1,000 ng/ml) or Sir (0.2, 2, 10, or 20 ng/ml) was added at day 0. On day 5, cells were harvested and analyzed by flow cytometry. OKT3 was used in this assay since both MAIT cells and non-MAIT cells can respond to this stimulus.

### Statistical Analysis

The Friedman test was used to detect differences across the four repeated matched time points after HSCT. If significant, the Bonferroni corrected Dunn’s post-test was used to detect differences between the time points. The Kruskal–Wallis test was used to detect differences between the patient samples and the HC, followed by Dunn’s post-test. To detect differences between two groups of paired samples, the Wilcoxon matched-pairs signed rank test was used. The Mann–Whitney test was used to detect differences between two groups of unpaired samples. The two-way ANOVA was used to detect differences between two groups over time. The Fisher’s exact test was used to detect differences between two groups with two factors of categorical data, and the Pearson’s chi-squared test when there were three or more factors. Tests were performed using GraphPad Prism 6 (GraphPad Software, La Jolla, CA, USA). Two-tailed analyses were used where appropriate, and an alpha value of <0.05 was considered significant.

## Results

### MAIT Cells Have a Delayed Reconstitution Pattern Compared to Non-MAIT T Cells

Mucosal-associated invariant T cell reconstitution after HSCT was investigated in 16 patients at 1–2, 3–6, 12, and 24 months post HSCT. The gating strategy is showed in Figure 1A. The proportion of MAIT cells within total T cells was approximately 10 times lower compared to the HC throughout the observation period, and no increase in frequency was seen 2 years after transplantation (Figure 1B). The absolute number of MAIT cells did not increase until 2 years after the transplantation (Figure 1C), whereas the number of non-MAIT T cells from the same samples increased significantly during the first year (Figure 1D). Further comparing the reconstitution of number of MAIT cells with non-MAIT T cells using a two-way ANOVA, we observed a significant difference in reconstitution of cell number over time between the two cell types (Figure S1A in Supplementary Material). Thus, the MAIT cell reconstitution started between the first and second year after HSCT, whereas the non-MAIT T cells showed a more linear reconstitution pattern over time, which seemed to quantitatively have reached a plateau after the first year.

To further investigate MAIT cell reconstitution, MAIT and T cell reconstitution at 24 months post HSCT was compared between the following groups: age (above or below median), treatment with anti-thymocyte globulin (ATG, yes/no), conditioning regimen [reduced intensity conditioning (RIC)/myeloablative conditioning (MAC)], and sex (female/male). We observed no differences in MAIT cell frequency between the two age groups, or between females and males (Figure 1E). No correlation between MAIT cell frequency and age was observed (Figure S1B in Supplementary Material). However, the patients not treated with ATG, as well as the patients conditioned with MAC rather than RIC had significantly higher MAIT cell frequencies (Figure 1E). The same results were found when investigating the estimated number of MAIT cells/ml of blood (Figure 1F), whereas neither ATG nor conditioning regimen had any impact on non-MAIT T cell numbers at 24 months (Figure 1G). For these two factors, similar patterns were observed for MAIT cells over the entire observation period (Figures S1C–H in Supplementary Material). Furthermore, at 24 months, the three patients who did receive a MAC conditioning but no ATG had the highest proportion of MAIT cells within our patient cohort, and the first, second, and fourth highest absolute number of MAIT cells. Thus, patients who received a MAC conditioning, as well as those who did not receive ATG, had higher MAIT cell numbers, an observation not evident for the non-MAIT T cells.

Finally, we summarized the chimerism analysis of the CD3^+^ fraction from peripheral blood. Of the available data, all patients were >99.9% donor at 12 and 24 months, and the majority had undetectable amounts of recipient DNA (Figure 1H). The number of patients affected by common pathogens after HSCT is listed in Table S1 in Supplementary Material. We found no correlations between infection/re-activation of the different pathogens and frequency or number of MAIT cells or between re-activation of CMV and expression of either PD-1 or CD69 on either MAIT cells or non-MAIT T cells (data not shown).

### MAIT Cells Are Activated after HSCT, although the PD-1 Expression Remains Unaffected

**Figure 2:**
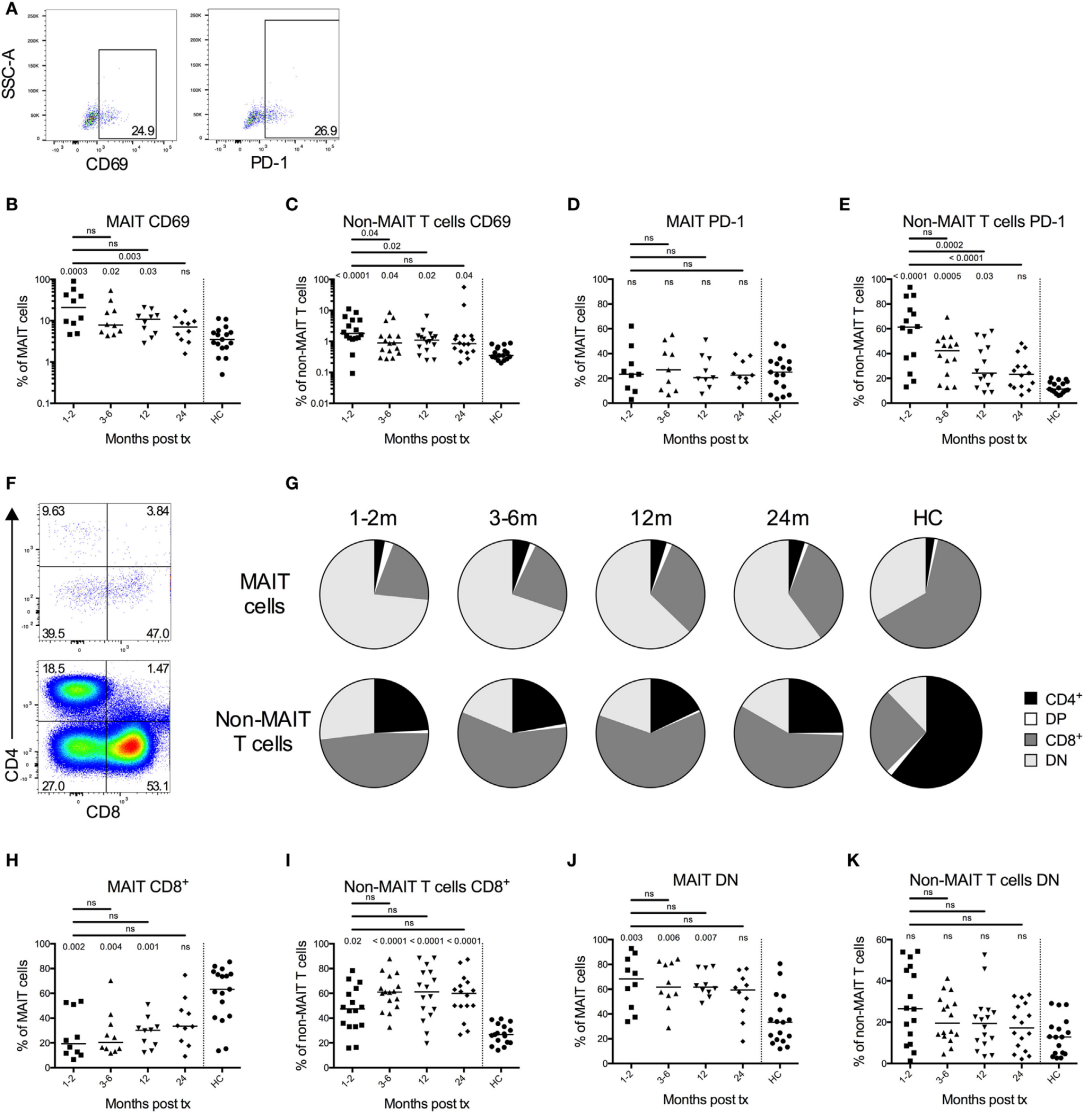
Mucosal-associated invariant T (MAIT) cells display an activated phenotype and altered CD4 and CD8 expression profile after hematopoietic stem cell transplantation (HSCT). **(A)** Representative flow cytometry plots from one patient sample showing the gating strategies for CD69 (left) and PD-1 (right) on MAIT cells. **(B)** CD69 expression on MAIT cells (*n* = 10) and **(C)** CD3^+^CD161^dim/neg^TCRVα7.2^dim/neg^ cells (non-MAIT T cells) (*n* = 16) examined at several time points after HSCT. **(D)** PD-1 expression on MAIT cells (*n* = 9) and **(E)** non-MAIT T cells (*n* = 14). Data from healthy controls (HC) are shown to the right of the dotted line [**(B–E)**, *n* = 17]. **(F)** Representative flow cytometry plots showing the CD4 and CD8 gating strategies of MAIT cells and non-MAIT T cells. **(G)** Pie charts with the median proportion of CD4^+^CD8^–^ (CD4), CD4^+^CD8^+^ (double positive, DP), CD4^–^CD8^+^ (CD8) and CD4^−^CD8^−^ (double negative, DN) MAIT cells (top, *n* = 10), and non-MAIT T cells (bottom, *n* = 16) at four time points after HSCT and in HC (*n* = 17). Proportion of **(H)** CD8^+^ MAIT cells (*n* = 10) and **(I)** CD8^+^ non-MAIT T cells (*n* = 16) at four time points after HSCT. Proportion of **(J)** DN MAIT cells (*n* = 10) and **(K)** DN non-MAIT T cells (*n* = 16) at four time points after HSCT. Data from HC are shown to the right of the dotted line [**(H–K)**, *n* = 17]. Horizontal lines indicate the median value. Comparisons were made using the Friedman test followed by Dunn’s post-test between the four time points after HSCT. The Kruskal–Wallis test followed by Dunn’s post-test was used to compare patients with controls. In **(B,C,H–K)**, numbers without bars above data points indicate *p*-values compared to HC.

To further characterize MAIT cell reconstitution, we examined the expression of the activation marker CD69 and the co-inhibitory marker PD-1. Representative plots on CD69 (left) and PD-1 (right) expression on MAIT cells from one patient sample is shown in Figure 2A. Significantly higher frequencies of MAIT cells expressed CD69 compared to the controls throughout the first year post HSCT (Figure 2B). After 2 years, this difference was no longer apparent, and there was also a significant decline in CD69 expression between the 1- and 2-month samples and the 24-month samples (Figure 2B). A similar pattern was observed for the non-MAIT T cells, although the overall CD69 frequency was approximately 10 times lower than for the MAIT cells (Figure 2C). The increased activation status of the MAIT cells did not correspond to a subsequent increase in PD-1 expression, as no differences were discernable, neither over time nor in relation to the controls (Figure 2D). On the other hand, non-MAIT T cells expressed high levels of PD-1 early after transplantation, and there was a significant linear temporal decline over 2 years (Figure 2E). The expression of CD69 as well as PD-1 on MAIT and non-MAIT T cells was not influenced by conditioning regimen (Figures S2A–D in Supplementary Material).

It has been shown that MAIT cells are predominantly CD8^+^ in healthy donors (19). A representative staining of CD4 and CD8 expression in MAIT cells (top) and non-MAIT T cells (bottom) from a patient sample is shown in Figure 2F. We observed that the proportion of CD8^+^ MAIT cells was significantly lower during the first year after HSCT compared to HC (Figures 2G,H). Consequently, the MAIT cells in HSCT patients were dominated by a DN phenotype (Figures 2G,J). In line with previous studies, the non-MAIT T cells in HSCT patients were on the other hand CD8^+^ to a larger extent compared to HC (Figures 2G,I). No differences in DN non-MAIT T cells were observed between patients and HC (Figure 2K). The frequency of CD8^+^ and DN MAIT cells did not differ significantly depending on conditioning regimen, although there was a trend toward that patients conditioned with MAC had a higher proportion of CD8^+^ MAIT cells at 24 months (*p* = 0.052) (Figures S2E,F in Supplementary Material).

### Impaired Responsiveness of MAIT Cells Early after Transplantation

**Figure 3:**
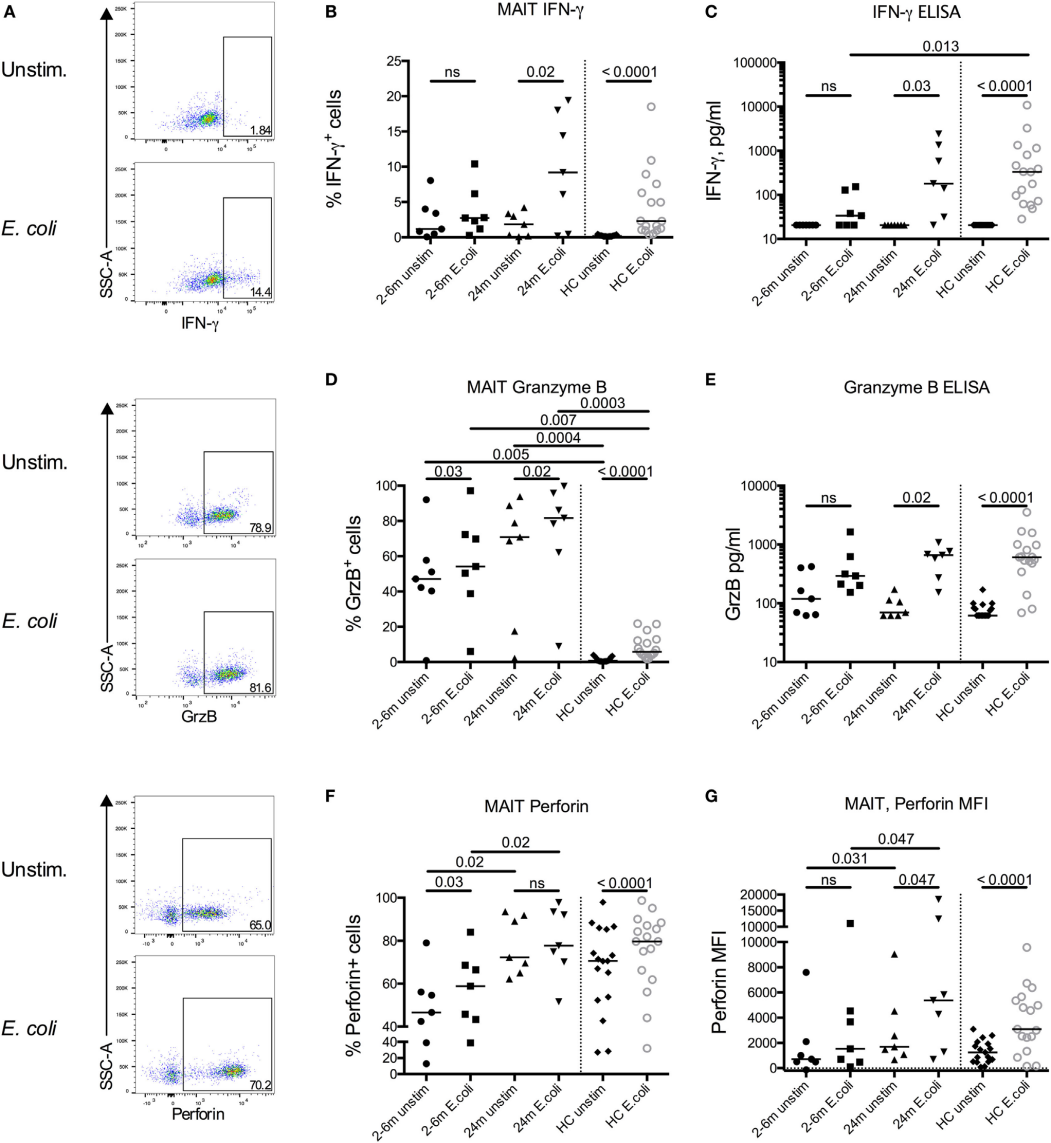
Impaired responsiveness of mucosal-associated invariant T (MAIT) cells early after transplantation. **(A)** Representative flow cytometry plots from the same patient sample showing the gating strategies for IFN-γ, granzyme B (GrzB), and perforin in MAIT cells. Intracellular expression of **(B)** IFN-γ, **(D)** GrzB, and **(F)** perforin in MAIT cells from hematopoietic stem cell transplantation patients in the absence (unstim) or presence of stimulation with *Escherichia coli* (*n* = 7). Levels of **(C)** IFN-γ and **(E)** GrzB in the supernatants from the same experiments as in **(B,D)** as analyzed by ELISA (*n* = 7). **(G)** Median fluorescence intensity (MFI) of perforin expression in MAIT cells (*n* = 7). Data from healthy controls (HC) are shown to the right of the dotted line [**(B–G)** *n* = 17]. Horizontal lines indicate median values. Wilcoxon’s test was used for comparisons between paired unstimulated and stimulated samples, as well as between paired patient samples from different time points. Comparisons between patient samples and healthy controls were made using Kruskal–Wallis test followed by Dunn’s post-test. ns, not significant.

To determine MAIT cell responsiveness to bacterial stimulation, PBMCs were stimulated with *E. coli* for 16 h, and intracellular expression of IFN-γ, GrzB, and perforin was determined by flow cytometry. Representative plots on expression of the respective markers on MAIT cells from an unstimulated and an *E. coli* stimulated patient sample are shown in Figure 3A. Patient samples from either 2, 3, or 6 months after transplantation were compared with the paired 24-month samples. MAIT cells from patients up to 6 months after HSCT showed no significant increase in expression of IFN-γ after *E. coli* stimulation (Figure 3B) and no significant secretion of IFN-γ was observed in supernatants from stimulated PBMCs (Figure 3C). However, at 24 months after HSCT the proportion of MAIT cells secreting IFN-γ in response to stimulation, as well as secreted levels of IFN-γ from PBMCs, were comparable to HC (Figures 3B,C). Non-MAIT CD4^+^ and CD8^+^ T cells had no or low expression of IFN-γ in response to *E. coli* stimulation in both patients and HC (Figures S3A,B in Supplementary Material).

Granzyme B was highly expressed intracellularly by MAIT cells in both the early and late patient samples even in the absence of stimulation (Figure 3D). This contrasted to HC MAIT cells, which did not express GrzB in a resting state, but intracellular expression was induced after stimulation (Figure 3D). When analyzing secreted levels of GrzB from PBMCs, we found that GrzB was not significantly released upon *E. coli* stimulation in samples acquired up to 6 months post HSCT, whereas the 24-month patient samples responded similarly compared to HC (Figure 3E). In line with the intracellular GrzB expression pattern in MAIT cells, a larger proportion of non-MAIT CD4^+^ and CD8^+^ T cells expressed intracellular GrzB in HSCT patients compared to HC, but the increase upon bacterial stimulation was absent or low (Figures S3C,D in Supplementary Material).

In their resting state, most MAIT cells express perforin (29), a finding confirmed in our control samples where a median of 71% of MAIT cells were positive for perforin in unstimulated conditions, and significantly increased after stimulation (Figure 3F). Similar proportions were observed in the MAIT cells from the 24-month samples. However, the expression of perforin was significantly lower in the patient samples from the first 6 months compared to 24 months, both in unstimulated and stimulated conditions (Figure 3F). A similar pattern was observed when examining the median fluorescence intensity of perforin expression in MAIT cells (Figure 3G). Non-MAIT CD4^+^ T cells had a low perforin expression, whereas the expression in CD8^+^ T cells was closer to that of the MAIT cells, and the response to bacterial stimulation was low or absent (Figures S3E,F in Supplementary Material).

### MAIT Cell Frequency Does Not Correlate with GvHD Status following HSCT

**Figure 4:**
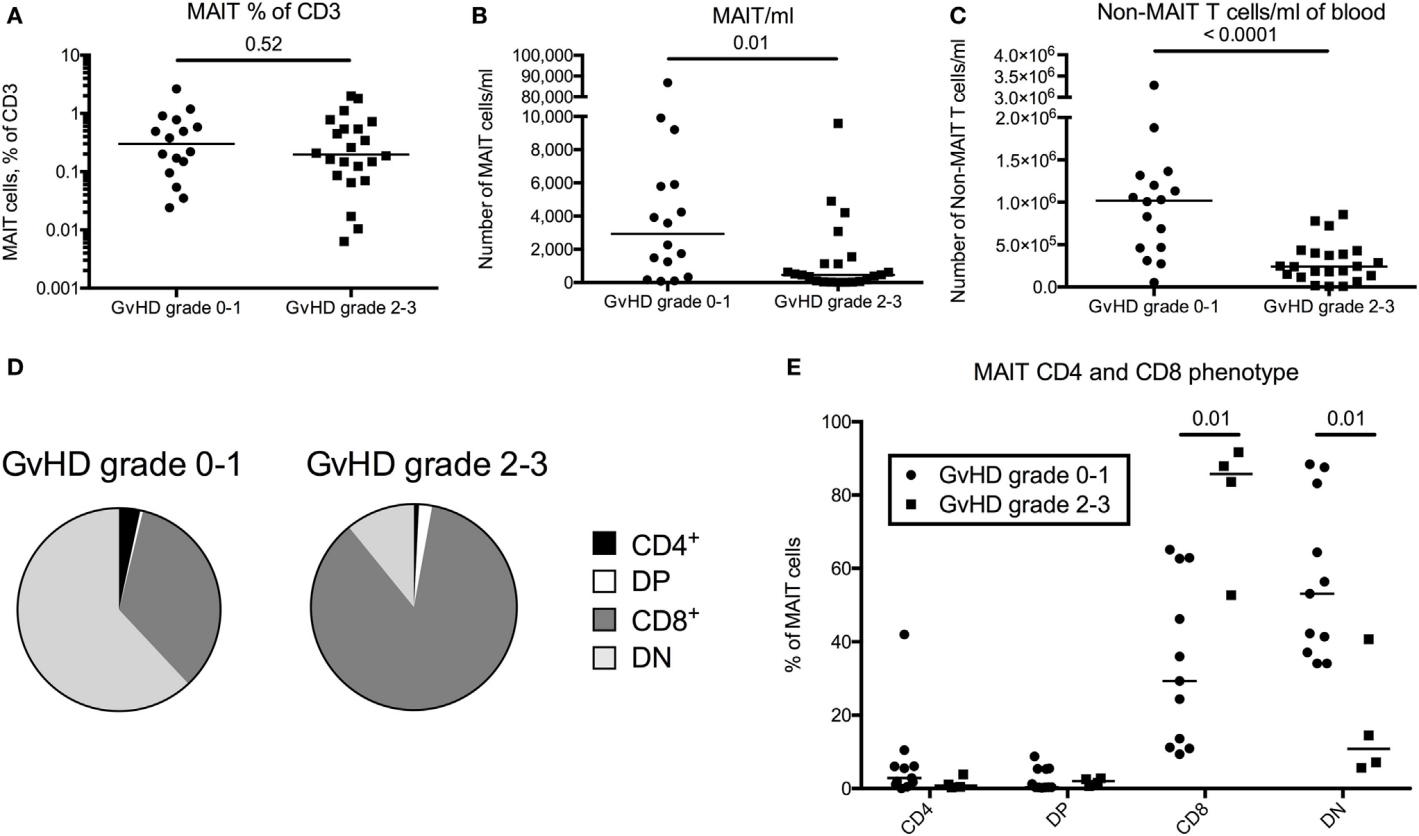
Mucosal-associated invariant T (MAIT) cell frequency does not correlate with graft-versus-host disease (GvHD) status following hematopoietic stem cell transplantation. **(A)** MAIT cells expressed as percentage of CD3^+^ cells, **(B)** number of MAIT cells/ml of blood, and **(C)** number of CD3^+^CD161^dim/neg^TCRVα7.2^dim/neg^ cells (non-MAIT T cells)/ml of blood compared between patients with GvHD grade 0–1 (*n* = 16) and grade 2–3 (*n* = 22). **(D)** Pie charts with the median proportion of CD4^+^CD8^−^ (CD4), CD4^+^CD8^+^ (double positive, DP), CD4^−^CD8^+^ (CD8), and CD4^−^CD8^−^ (double negative, DN) MAIT cells from patients with GvHD grade 0–1 (*n* = 11) and 2–3 (*n* = 4). **(E)** Proportions of CD4^+^, DP, CD8^+^, or DN MAIT cells compared between patients with GvHD grade 0–1 (*n* = 11) and 2–3 (*n* = 4). Horizontal lines in dot plots indicate the median value. Comparisons were made using the Mann–Whitney test.

T cell reconstitution is impaired in patients with acute GvHD and is linked to impaired thymic output (30). To investigate a potential correlation between acute GvHD and reconstitution of MAIT cells, we included data on 22 patients with acute GvHD grade 2–3. All patients received corticosteroids, and the samples were collected early after diagnosis at a median of 3.3 months (range 0.5–13.3 months) after transplantation, hence the comparison was made with the 3-month samples from the GvHD grade 0–1 group (*n* = 17). When analyzing the proportion of MAIT cells within CD3^+^ T cells, no difference was observed between the patients with acute GvHD grade 2–3 and the patients with grade 0–1 (*p* = 0.52, Figure 4A). When calculating the estimated number of cells/ml, the number of MAIT cells was a median of 6.4-fold lower in patients with GvHD grade 2–3 than in the patients with grade 0–1 (Figure 4B). As expected, the non-MAIT T cells showed a similar pattern (Figure 4C), indicating that neither MAIT cell number nor frequency could be correlated to GvHD status. However, in contrast to the domination of DN MAIT cells in the patient cohort devoid of GvHD, the MAIT cells in acute GvHD patients predominantly expressed CD8 (Figures 4D,E).

### MAIT Cells Are More Sensitive to Immunosuppressive Drugs than Non-MAIT T Cells *In Vitro*

**Figure 5:**
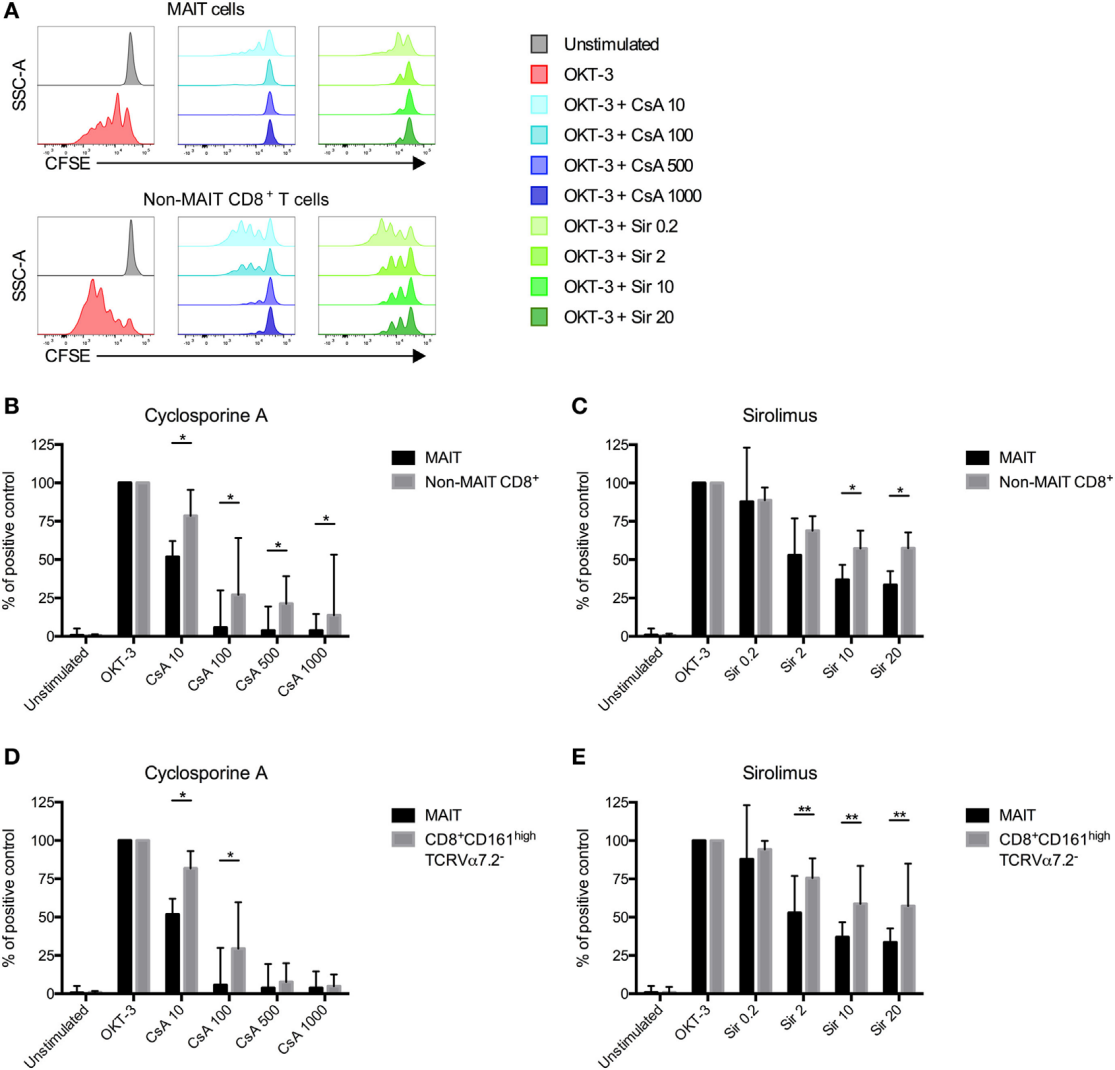
Mucosal-associated invariant T (MAIT) cells are more sensitive to immunosuppressive drugs than non-MAIT T cells. **(A)** Representative flow cytometry histograms from one healthy donor showing the CFSE expression in MAIT cells and CD3^+^CD161^dim/neg^TCRVα7.2^dim/neg^ (non-MAIT) CD8^+^ T cells. CFSE-labeled peripheral blood mononuclear cells from healthy controls were stimulated with OKT-3 in the absence of presence of indicated concentrations of **(B)** cyclosporine A (CsA) (*n* = 7) and **(C)** sirolimus (Sir) (*n* = 8). After 5 days of culture, cells were harvested and stained for flow cytometry. Data were acquired for MAIT cells and non-MAIT CD8^+^ T cells and presented as the percentage of CFSE^low^ cells after stimulation with OKT-3 only. **(D,E)** Same as in **(B,C)**, but comparisons in proliferation were made between MAIT cells and CD8^+^CD161^high^TCRVα7.2^−^ T cells (*n* = 7 and 8, respectively). Interleaved bars show median and the upper range of paired samples. Comparisons between paired samples were made using the Wilcoxon test. *p < 0.05, **p < 0.01.

Following HSCT, all patients receive immunosuppressive drugs as GvHD prophylaxis. Common protocols include either CsA or Sir (31). Immunosuppression is usually tapered and ended around 6 months after transplantation. As shown in Figure 1C, the total number of MAIT cells/ml of blood started to increase between 12 and 24 months after transplantation. As this coincides with the discontinuation of immunosuppressive drugs, we investigated the effect of CsA and Sir on MAIT cell proliferation. By labeling cells from HC with CFSE, we examined the proliferation in response to OKT-3 stimulation in the presence or absence of clinically relevant, as well as higher concentrations of the drugs. We have previously confirmed that the drug concentrations are stable over 6 days of *in vitro* culture (32). MAIT cells proliferated strongly in response to OKT-3 stimulation (Figure 5A). Adding titrating levels of either CsA or Sir induced a potent suppression of proliferation on both MAIT and non-MAIT CD8^+^ T cells (Figure 5A). When comparing MAIT cells with the non-MAIT CD8^+^ T cells from the same cultures, it was revealed that both CsA and Sir had a stronger suppressive effect on the MAIT cells (Figures 5B,C). However, no significant correlation between treatment duration of immunosuppressive drugs and proportion of MAIT cells at 24 months could be observed (Figure S4A in Supplementary Material). CD8^+^CD161^high^ cells have previously been shown to be less susceptible to immunosuppressive drugs than other T cell subsets (33). Comparing the MAIT cells to CD8^+^CD161^high^Vα7.2^−^ cells (excluding MAIT cells), we found the latter to be less sensitive to immunosuppression than MAIT cells, using both CsA (Figure 5D) and Sir (Figure 5E). The CD8^+^CD161^high^Vα7.2^−^ cells were also less sensitive than the remaining non-MAIT T cells (Figures S4B,C in Supplementary Material). Dividing the MAIT cells into CD4^+^, DN, and CD8^+^, we found that almost all of the CD4^+^ MAIT cells proliferated in response to the stimulation, compared to 55% of the CD8^+^, and 35% of the DN MAIT cells. For the non-MAIT T cells, both CD4^+^ and CD8^+^ cells proliferated well, but the proliferation of DN T cells was impaired (Figures S4D,E in Supplementary Material).

## Discussion

Patients undergoing HSCT are critically dependent on the reconstitution of the immune system for their survival. Systemic MAIT cell deficiency have been correlated with severe systemic diseases such as tuberculosis (19, 34), hepatitis C (35), and HIV (36), but also with septic as well as non-septic critically ill patients (37). We show a severely delayed reconstitution of MAIT cells following HSCT. Not even 2 years after the transplantation have the MAIT cells started to increase toward normal proportions within the T cell population. Out of the few MAIT cells in the circulation, the response to bacterial stimulus was impaired during the first 6 months after transplantation, but normalized in the 24-month samples, providing evidence of a slow functional reconstitution.

We actively selected patients with as few major post-transplantation complications as possible. By this, we aimed at minimizing known major confounding factors of immune reconstitution, namely severe acute or chronic GvHD and relapse. In addition, all of the 17 patients selected were alive 2 years after HSCT. Based on our present knowledge, this should result in our data being a “best case scenario” from a reconstitution perspective for adult HSCT patients. The drawback of such a study design is that it does not support the analysis of the impact of these complications on MAIT cell reconstitution. Although the total amount of analyzed samples was high, we were limited by the low number of patients available for subanalysis. The patient group was also heterogeneous in terms of characteristics, and based on the conscious selection bias described earlier. It would be of great interest to follow a new cohort of patients prospectively and include more patients, but with fewer time points for sample collection. By increasing the number of patients, multivariate statistics could be performed, and the impact of factors such as conditioning, ATG use, acute and chronic GvHD, relapse, bacterial, fungal, and viral infection could be discerned with more statistical power.

MAIT cells as percentage of T cells did not increase throughout the 2-year observation period and was consequently significantly lower than HC (Figure 1B). The absolute number of MAIT cells/ml of blood only increased 2 years after transplantation, whereas the non-MAIT T cells reached a stable plateau phase at 1 year (Figures 1C,D). This together with the results from the two-way ANOVA (Figure S1A in Supplementary Material) suggests that MAIT cell reconstitution is slower compared to non-MAIT T cells. Although we could not perform chimerism analysis on the MAIT cells, data on total CD3^+^ cells indicated that the investigated MAIT cells were of donor origin, at least at the later time points (Figure 1H). During the revision of this manuscript, Bhattacharyya et al. published a study on peripheral MAIT cells after allogeneic HSCT. They found that the majority of peripheral blood MAIT cells were of donor origin after HSCT at 1 and 3 months, further supporting the donor origin of MAIT cells. However, the chimerism of mucosal MAIT cells after HSCT remains unknown, and it would be of great interest to study the origin of these MAIT cells.

Abrahamsson et al. investigated reconstitution of CD8^+^ MAIT cells in a small cohort of patients following autologous HSCT for multiple sclerosis (MS) (38). The relative frequency of MAIT cells dropped after the transplantation and remained low throughout the 2-year observation period, but it should be noted that only CD8^+^ MAIT cells were included in their analysis. Novak et al. examined MAIT cell reconstitution in 29 patients undergoing autologous HSCT for malignant diseases (39). During the observation period of 100 days, the relative MAIT cell frequency remained below normal levels, although for one-third of the patients, measuring total number of MAIT cells, a trend toward normalization was observed. Autologous and allogeneic stem cell transplantation differ strongly from an immune reconstitution standpoint, mostly because of the GvHD/GvL effect and the immunosuppression. Because of this, it is intriguing that our findings partly coincide with the above mentioned studies in autologous HSCT.

One reason for the slow reconstitution could be the relatively old age of the patients in our study. Novak et al. showed a negative correlation of MAIT cells with age, both in total number of MAIT cells as well as percentage of total T cells (40). MAIT cells and non-MAIT T cells depend on a functional thymus for development (26, 27). It is also known that thymic function is strongly correlated with a successful immune reconstitution (41, 42), and higher age negatively correlates with thymic output in HSCT patients (12). However, we did not find any significant correlation between MAIT cell reconstitution and age (Figures 1E,F; Figure S1B in Supplementary Material), indicating that other factors than age and thymic function influence MAIT cell reconstitution. It would also have been of interest to study to which extent the MAIT cells were *de novo* generated from thymus and expanded *in vivo* from the graft. Unfortunately, TREC analysis was not available for MAIT cells in these patients. Bhattacharyya et al. examined MAIT cells clonotypes after transplantation, which indicated that at least some MAIT cell clonotypes were expanded *in vivo* from the graft (43).

We observed that patients who received MAC conditioning, as well as those that did not receive ATG had significantly higher number of MAIT cells, but not non-MAIT T cells, 2 years post HSCT (Figures 1E–G). Five out of six patients in the MAC group received cyclophosphamide as part of their conditioning. Since cyclophosphamide mainly targets activated T cells (44), and given that the MAIT cells were highly activated early after transplantation, the higher number of MAIT cells in the MAC patients were surprising. However, Abrahamsson et al. also found that patients treated with cyclophosphamide for MS had significantly higher frequencies of MAIT cells 6–24 months following therapy than patients treated with alemtuzumab (38). Alemtuzumab targets CD52, thus effectively affecting lymphocytes, in a manner similar to ATG. Thus, our findings in large confirm the observations by Abrahamsson et al. that MAIT cells are seemingly more sensitive to direct targeting by monoclonal antibodies, than to broad cytotoxic chemotherapy treatments. The common denominator of the patients treated with RIC was the use of the chemotherapeutic drug fludarabine, which was given to all RIC patients. Certain drug metabolites have been shown to regulate the function of MAIT cells (45), and it would be of great interest to elucidate whether particular chemotherapeutic drugs affect MAIT cells more than others.

Early after HSCT, the MAIT cells expressed increased levels of the early activation marker CD69. This expression gradually declined over the following 2 years until it reached levels similar to that of HC (Figure 2B). Others have correlated an increase in CD69 expression on MAIT cells with systemic lupus erythematosus (46) and ulcerative colitis (47). CD69 has also been confirmed as a reliable marker of MAIT cell response to bacterial stimuli (19). Interpreting our findings in this context, the increased levels of CD69 early after HSCT could be due to the general pro-inflammatory state, as well as due to the recurrent bacterial infections occurring during the first 6 months after transplantation. In line with our observations, Bhattacharyya et al. also found that peripheral MAIT cells expressed high levels of CD69 early after transplantation and they also speculated that it could be due to elevated cytokine levels (43). Further studies are needed to evaluate the causes of an increase in CD69 expression in systemic MAIT cells.

PD-1 impairs the IFN-γ production in MAIT cells (48), and an increased PD-1 expression on MAIT cells has been associated with diseases, such as tuberculosis (48, 49), HIV (50), and hepatitis C (35). We did not observe any fluctuations in PD-1 expression on MAIT cells following HSCT, and the levels did not differ from that of HC (Figure 2D). In contrast, approximately 60% of the non-MAIT T cells expressed PD-1 during the first months after HSCT. This was followed by a gradual decrease, until normal levels at 2 years (Figure 2E). Kinter et al. showed that PD-1 expression is increased in response to the cytokines sharing the common γ-chain (IL-2, IL-7, IL-15, and IL-21) (51), cytokines known to be increased at this time point after transplantation (8) as well as to have a strong impact on T cell proliferation in HSCT (9). Thus, the highly increased levels of PD-1 can probably at least partly be explained by the effect of the cytokines sharing the common γ-chain. As the non-MAIT T cells do expand, this may lead to increased levels of PD-1 expression during the first year after HSCT, whereas the MAIT cells do not proliferate and thereby express normal levels of PD-1. The high PD-1 expression on T cells during the first 12 months after transplantation could potentially in part contribute to the increased prevalence of opportunistic infections often observed in HSCT patients.

Another interesting finding was that the MAIT cells in HSCT patients had a different expression pattern of CD4 and CD8 compared to HC MAIT cells (19). In HC the majority of MAIT cells were CD8^+^, whereas in the HSCT patients early after transplant almost 75% were DN (Figure 2G). This difference was significant up until 12 months, but not for the 24-month sample, indicating a gradual normalization (Figures 2H,J). In the autologous transplant setting, Novak et al. noted that the proportion of CD8^+^ MAIT cells declined early after transplantation, but that it normalized already after 2 months (39), which contrasts to our findings in allogeneic HSCT. It is known that non-MAIT T cells express an inverted CD4^+^/CD8^+^-ratio long after allogeneic HSCT (52), a finding supported by our data (Figures 2G,I,K). Novak et al. examined changes in the ratio of DN/CD8^+^ MAIT cells in individuals between 0 and 100 years of age (40). They found that the expression of CD8 was negatively correlated with age, and that the frequency drops below 50% in the oldest individuals. The causes for this remains unknown, but it could be speculated that the same factors influence the MAIT cells in our patient cohort.

We examined MAIT cell responsiveness to *E. coli* stimulation in the HSCT patients. The response to the stimuli was impaired in the samples from 2 to 6 months after HSCT, but the 24-month samples were in line with those from HC when measuring IFN-γ expression as well as secretion (Figures 3B,C). Others have shown that CD8^+^ MAIT cells respond more potently with secretion of IFN-γ, TNF-α, and GrzB upon stimulation with PMA/ionomycin than DN MAIT cells (53), an observation that could explain the poor response of MAIT cells early after transplantation since the majority of MAIT cells were DN. In this study, the number of MAIT cells in HSCT patients was too low to allow delineation of CD8^+^ and DN MAIT cell to compare responses. However, we did not observe any difference in response from CD8^+^ and DN MAIT cells in the HC (data not shown). Perforin expression was lower in the early samples post HSCT compared to the 24-month samples (Figure 3F). In contrast, the intracellular expression of GrzB was higher both in unstimulated conditions and after stimulation compared to the HC up to 2 years after transplantation (Figure 3D). This was not only confined to MAIT cells, since non-MAIT T cells showed a very similar pattern. The reasons for this increase in background expression of GrzB are unknown. Leeansyah et al. showed a strong induction of GrzB by stimulation with IL-7 alone (21), and it could be speculated that the known increased plasma levels of this cytokine in HSCT patients has induced a background expression of GrzB.

As acute GvHD is known to negatively impact thymic output and immune reconstitution (30, 41), and the severity of chronic GvHD has been correlated to decreased frequency of MAIT cells (54), we included data on MAIT cell proportions and number/ml of blood from a cohort of patients diagnosed with acute GvHD grade 2–3. As expected, the total number of non-MAIT T cell was significantly decreased in these patients (Figure 4C), probably due to a general immunosuppressive effect of the GvHD as well as the corticosteroid treatment. However, acute GvHD grade 2–3 did not appear to affect MAIT cell frequency (Figure 4A), as the decrease in the number of MAIT cells was proportionate to that of the non-MAIT T cells in these patients (Figures 4A–C). In line with our observations, Bhattacharyya et al. observed a drop in MAIT cell number at 1 month after HSCT in patients who later developed acute GvHD (43). Stikvoort et al. found that an increased severity of chronic GvHD was correlated to lower frequencies of MAIT cells (54). Acute and chronic GvHD have different modes of action and appear to affect MAIT cells in different ways. It would be of interest to further study how MAIT cells are affected by acute and chronic GvHD to understand the varying detrimental effect on the immune reconstitution seen in these diseases. We also found that the acute GvHD patients had a higher proportion of circulating CD8^+^ MAIT cells compared to the patient cohort without GvHD (Figures 4D,E). The reason for this is unclear, but since CD8^+^ MAIT cells had a higher proliferative capacity compared to DN MAIT cells (Figure S4D in Supplementary Material), it can be speculated that the cytokine storm in GvHD patients might have contributed to an expansion of CD8^+^ MAIT cells at the expense of DN MAIT cells.

Recent studies have shown that MAIT cells utilize heterogenic T cell receptor repertoires in their response to different microbes, and that these clones have a dissimilar response to bacterial stimulation (55, 56). It would be of great interest to investigate whether CD8^+^ and DN MAIT cells differ in terms of T cell receptor diversity, and subsequent antigen specificity and proliferative as well as effector potential.

The reconstitution of CD8^+^CD161^high^ cells, of which a proportion presumably were MAIT cells (24), was investigated by van der Waart et al. for up until 12 months after HSCT (33). They observed a gradual decrease until 6 months when the levels stabilized at low levels. Furthermore, they found an increase in mRNA expression of the ABCB1 multi-drug transporter protein in this cell population, and an increased ability to transport the target dye Rhodamine 123 over the cell membrane, and that that the CD8^+^CD161^high^ cells were less suppressed by CsA compared to the other T cells. In contrast, we found that MAIT cells were more sensitive to immunosuppression than the remaining CD8^+^ T cells *in vitro* (Figures 5A–C). However, we could confirm that CD8^+^CD161^high^Vα7.2^−^ cells were less affected by the drugs than the MAIT cells (Figures 5D,E), as well as the other T cells, but not to the same extent as shown by van der Waart et al. (Figures S4B,C in Supplementary Material). One potential explanation of the conflicting results could be differences in our assays. They used allo-PBMCs, IL-2, IL-7, and IL-15 as stimulants, whereas we only stimulated with OKT-3. As CsA targets the intracellular pathway following IL-2 stimulation by binding calcineurin, the cells in their study were still fully stimulated by IL-7 and IL-15, whereas our only mode of stimuli was more directly targeted by the drug, probably resulting in a more potent suppression of proliferation in our cultures. Interpreting the findings from the *in vitro* results, one possible explanation of the delayed MAIT cell reconstitution could be an increased sensitivity to the immunosuppressive drugs used in routine care after HSCT. Indeed, the number of MAIT cells/ml of blood did more than double between the 3- and 6-month sample and the 24-month sample (Figure 1C). We found no significant correlation between the proportion of MAIT cells at 24 months and duration of treatment with immunosuppressive drugs (Figure S4A in Supplementary Material). However, it is interesting that the patient receiving a graft from a twin, and hence not treated with immunosuppressive drugs, had both the highest proportion and number of MAIT cells at 24 months.

It is known that the composition of the gastrointestinal microbiota is altered after allogeneic HSCT (57). All patients in our study were treated with ciprofloxacin at the time of transplantation and trimethoprim/sulfamethoxazole following transplantation, further predisposing for a disturbed bacterial diversity. Thus, it is likely that a poor microbial composition may affect MAIT cell development, similar to that observed in germ-free mice (17). Interestingly, Bhattacharyya et al. found that intestinal colonization with certain bacteria, namely *Blautia* species and *Bifidobacterium longum*, was correlated with higher peripheral MAIT cell numbers (43). It remains to be determined if these species specifically contribute to TCR-mediated proliferation of MAIT cells, or whether they are surrogate markers for a better microbial diversity. Another aspect is that the conditioning regimen and other treatment modalities can cause an inflammation in the intestinal tract, which potentially could recruit MAIT cells from the blood to the mucosa. We only followed MAIT cell reconstitution in peripheral blood and for future studies it will be important to examine mucosal MAIT cells after HSCT.

In conclusion, we have investigated the reconstitution of MAIT cells in allogeneic HSCT patients. Compared to non-MAIT T cells, the MAIT cell reconstitution was severely delayed, but the conditioning regimen appeared to influence the outcome. Acute GvHD was not correlated with a decreased frequency of MAIT cells, although differences in MAIT cell phenotype could be detected. The MAIT cells had an impaired response to bacterial stimulation during the first months, but normalized 2 years post HSCT. The delayed reconstitution of MAIT cells could in part be explained by an increased sensitivity to common immunosuppressive drugs. Further studies on MAIT cells after allogeneic HSCT are needed to further elucidate the factors influencing their reconstitution as well as their role in immunity after HSCT.

## Ethics Statement

Ethical approval was obtained from the regional review board of ethics in research of Karolinska Institutet (2010/760-31/1, 2010/452-31/4, 2014/2132-32). Written informed consent was received from participants before inclusion in the study.

## Author Contributions

MS conceived, designed, performed, and analyzed the experiments, interpreted the results, performed statistical analysis, and wrote the paper. TE conceived, designed, and performed the experiments and interpreted the results. LG designed, performed, and analyzed the experiments. TP designed the experiments. MR designed the experiments and interpreted the results. IM interpreted the results and wrote the paper. HK conceived, designed, and analyzed the experiments, interpreted the results, and wrote the paper. All authors participated in final approval of the manuscript.

## Conflict of Interest Statement

The authors declare that the research was conducted in the absence of any commercial or financial relationships that could be construed as a potential conflict of interest.
