# Supplementary material for: Mucosal-Associated Invariant T Cells Display a Poor Reconstitution and Altered Phenotype after Allogeneic Hematopoietic Stem Cell Transplantation
Source: Front Immunol. 2017 Dec 21;8:1861. doi: 10.3389/fimmu.2017.01861 (PMC5742569; doi:10.3389/fimmu.2017.01861)
Supplement: Supplementary file 1 [file Table_1.PDF]

Supplementary Table S1. Number of patients infected or re-activated with the indicated pathogen(s)

	**Yes**	**No**
Bacterial blood stream infection	2	15
CMV (blood)	10	7
EBV (blood)	3	14
Herpes simplex virus (skin)	3	14
Varicella zoster virus (skin)	3	14
Candida albicans (oral cavity)	3	14
