# Supplementary material for: Mucosal-Associated Invariant T Cells Display a Poor Reconstitution and Altered Phenotype after Allogeneic Hematopoietic Stem Cell Transplantation
Source: Front Immunol. 2017 Dec 21;8:1861. doi: 10.3389/fimmu.2017.01861 (PMC5742569; doi:10.3389/fimmu.2017.01861)
Supplement: Supplementary file 2 [file Image_1.PDF]

Supplementary figure S1

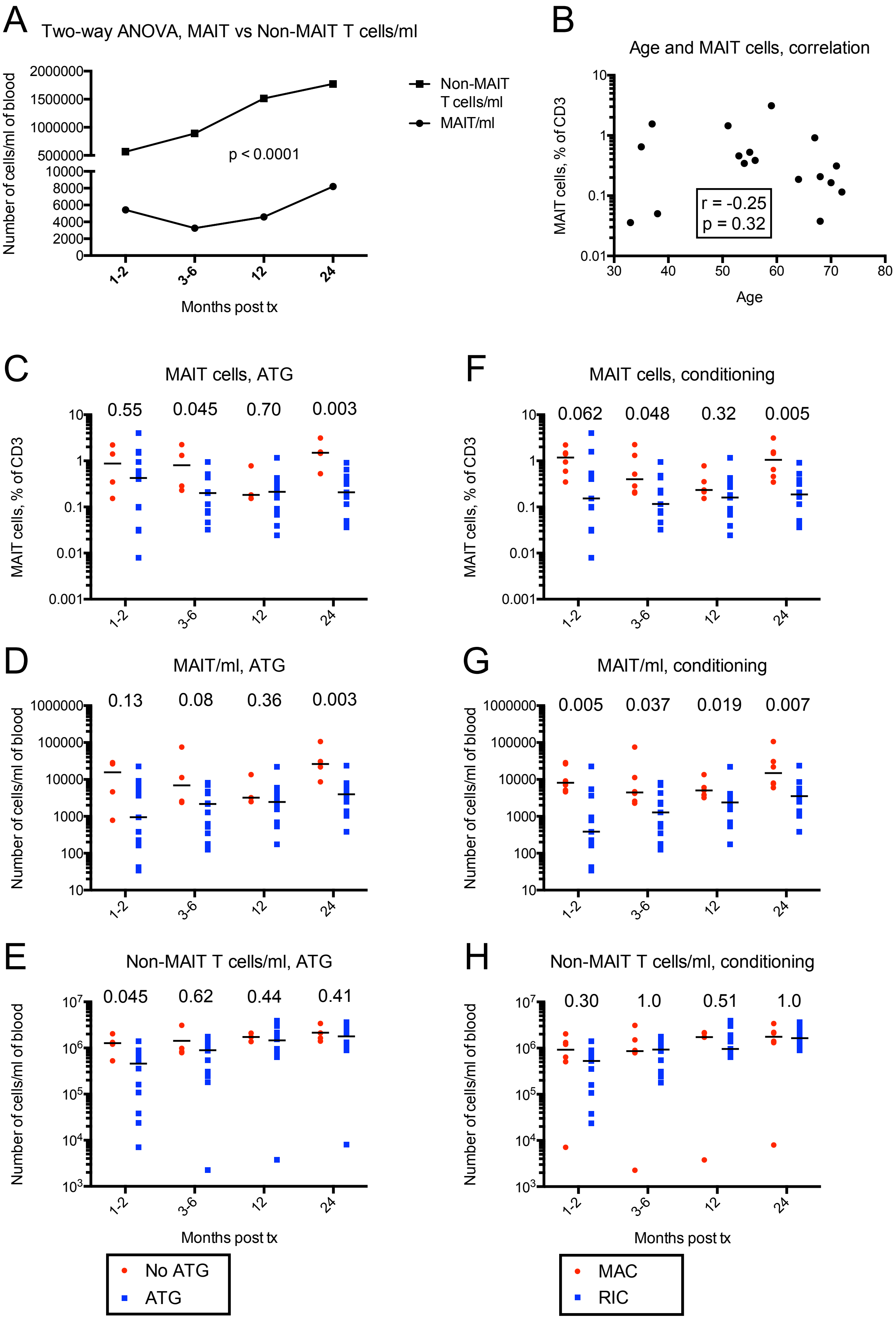

Supplementary figure S1. MAIT cell reconstitution differs from that of non-MAIT T cells and is influenced by conditioning regimen (A) Number of MAIT cells and non-MAIT T cells per ml of blood from the same samples paired over time. Symbols indicates mean value of samples. Comparison was made using the two-way ANOVA to discern an interaction between the factors cell type and time. Although the data was non-parametric, the two-way ANOVA complement the analysis in Figure 1. **(B)** MAIT cell proportion did not correlate to the age of the patients, as analyzed by Spearman's rank correlation test. **(C)** MAIT cells as percentage of $CD3^+$ cells, **(D)** number of MAIT cells per ml blood and **(E)** number of non-MAIT T cells per ml of blood at four time points after HSCT in patients not treated with ATG (n = 4, except for the 12 months time point where n = 3) and in patients treated with ATG (n = 13). **(F)** MAIT cells as proportion of $CD3^+$ cells, (G) number of MAIT cells per ml blood, and (H) number of non-MAIT T cells per ml blood at four time points after HSCT divided into patients who received myeloablative conditioning (MAC, n = 6, except for the 12 months time point where n = 5) and patients who received reduced intensity conditioning (RIC, n = 11). Horizontal lines in dot plots indicate median values. Comparisons between different groups in **(C-H)** were made using the non-parametric Mann-Whitney test.
