# Supplementary material for: Mucosal-Associated Invariant T Cells Display a Poor Reconstitution and Altered Phenotype after Allogeneic Hematopoietic Stem Cell Transplantation
Source: Front Immunol. 2017 Dec 21;8:1861. doi: 10.3389/fimmu.2017.01861 (PMC5742569; doi:10.3389/fimmu.2017.01861)
Supplement: Supplementary file 3 [file Image_2.PDF]

Supplementary figure S2

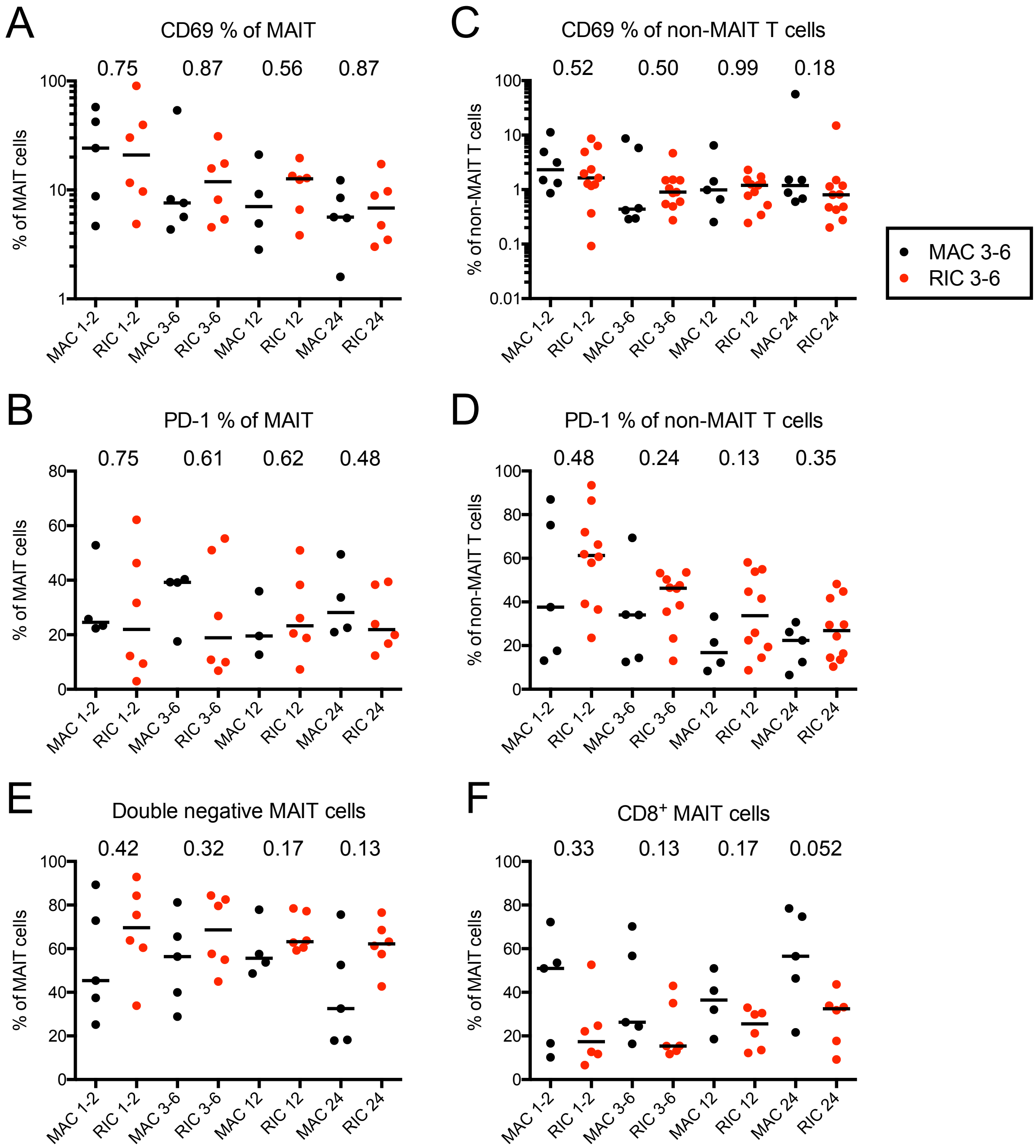

Supplementary figure S2. Effect on conditioning regimen on expression of cell surface molecules. Expression of **(A)** CD69 and **(B)** PD-1 on MAIT cells at indicated time points after HSCT, divided into patients who received myeloablative conditioning (MAC, *n* = 5, except for the 12 months time point where *n* = 4) and patients who received reduced intensity conditioning (RIC, *n* = 6). Expression of **(C)** CD69 and **(D)** PD-1 on non-MAIT T cells at indicated time points after HSCT, divided into patients who received MAC (*n* = 6, except for the 12 months time point where *n* = 5) and patients who received RIC (*n* = 11). **(E)** Proportion of double negative ($CD4^-CD8^-$) and **(F)** $CD8^+$ MAIT cells at indicated time points after HSCT, divided into patients who received MAC (*n* = 5, except for the 12 months time point where *n* = 4) and patients who received RIC (*n* = 6).
