# Supplementary material for: Mucosal-Associated Invariant T Cells Display a Poor Reconstitution and Altered Phenotype after Allogeneic Hematopoietic Stem Cell Transplantation
Source: Front Immunol. 2017 Dec 21;8:1861. doi: 10.3389/fimmu.2017.01861 (PMC5742569; doi:10.3389/fimmu.2017.01861)
Supplement: Supplementary file 4 [file Image_3.PDF]

Supplementary figure S3

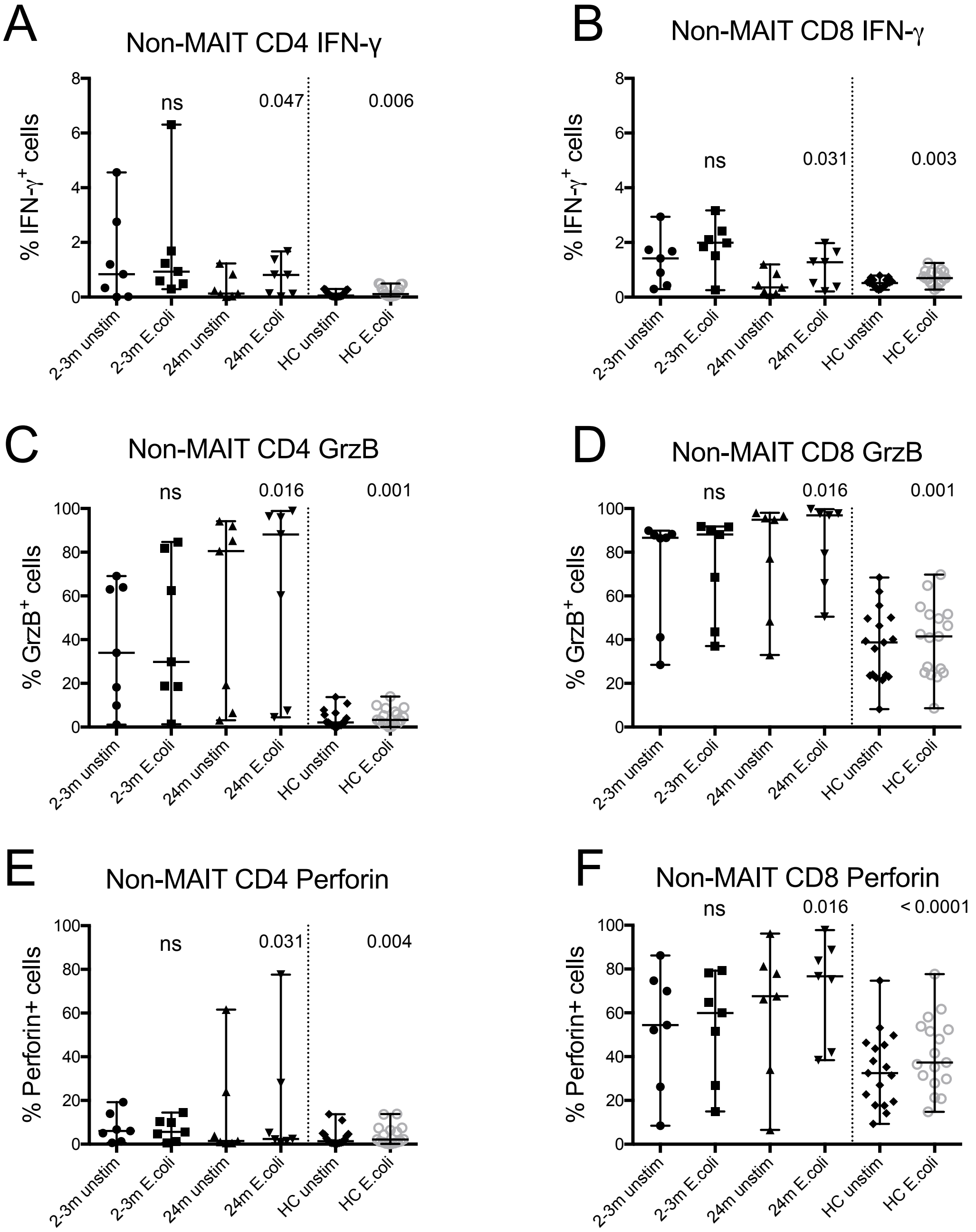

Supplementary figure S3. Expression of cytotoxic molecules in non-MAIT T cells after stimulation with E. coli. Intracellular expression of IFN-γ in **(A)** $CD4^+$ and **(B)** $CD8^+$ non-MAIT T cells in the absence (unstim) or presence of stimulation with *E. coli* at indicated time points after HSCT (n = 7). Intracellular expression of granzyme B (GrzB) in **(C)** $CD4^+$ and **(D)** $CD8^+$ non-MAIT T cells after stimulation with *E. coli* (n = 7). Intracellular expression of perforin in **(E)** $CD4^+$ and **(F)** $CD8^+$ non-MAIT T cells after stimulation with *E. coli* (n = 7). Data from healthy controls (HC) are shown to the right of the dotted line (**A-F** n = 17). Horizontal lines in dot plots indicate the median value. Comparisons between paired unstimulated and stimulated samples were made using the Wilcoxon test. Numbers indicate *P*-values for comparison between unstimulated and stimulated samples. ns = not significant.
