# Supplementary material for: Mucosal-Associated Invariant T Cells Display a Poor Reconstitution and Altered Phenotype after Allogeneic Hematopoietic Stem Cell Transplantation
Source: Front Immunol. 2017 Dec 21;8:1861. doi: 10.3389/fimmu.2017.01861 (PMC5742569; doi:10.3389/fimmu.2017.01861)
Supplement: Supplementary file 5 [file Image_4.PDF]

# Supplementary figure S4

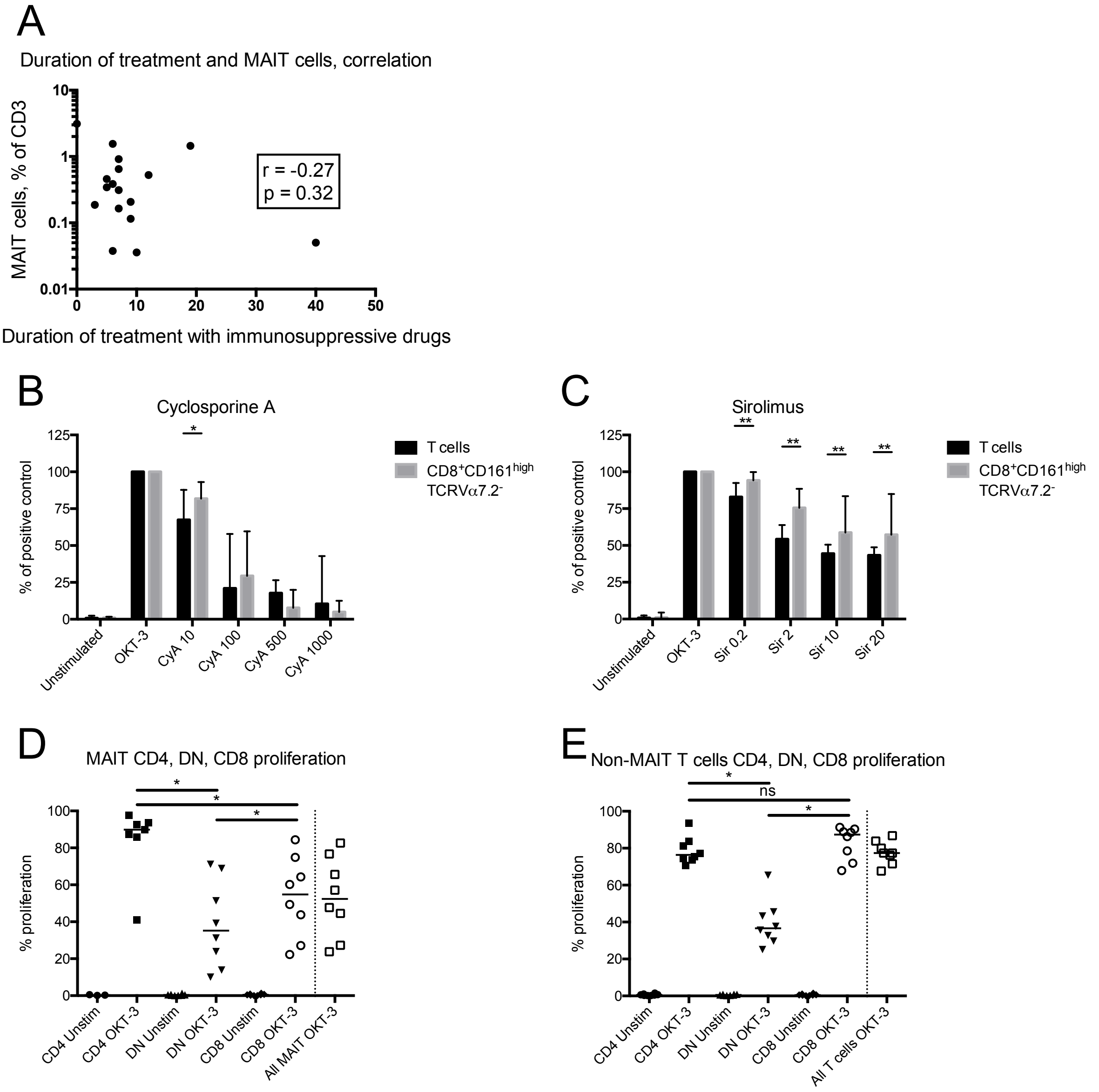


**Supplementary figure S4. Effects on immunosuppressive drugs on T cell subsets, and proliferative potential of MAIT and conventional T cells. (A)** MAIT cell proportion did not correlate to the duration of treatment with immunosuppressive drugs, as analyzed by Spearman's rank correlation test. CFSE-labeled PBMCs from healthy controls were stimulated with OKT-3 and indicated concentrations of **(B)** Cyclosporine A (CsA, (*n* = 7)) and **(C)** sirolimus (*n* = 8). After five days of culture, cells were harvested and stained for flow cytometry. Data was acquired for $CD8^+CD161^{high}TCRV\alpha7.2^-$ cells and non-MAIT T cells, and presented as the percentage of $CFSE^{low}$ cells after stimulation with OKT-3 only. Interleaved bars show median and the upper range of paired samples. Comparisons between paired samples were made using the Wilcoxon test. Proportion of $CFSE^{low}$ cells within the $CD4^+$, double negative (DN), and $CD8^+$ **(D)** MAIT cell and **(E)** non-MAIT T cell populations after stimulation with OKT3. Horizontal lines in dot plots indicate the median value. Comparisons were made using the Friedman test followed by the Wilcoxon test followed by Bonferroni correction. * $p < 0.05$, ** $p < 0.01$.
